# Neuromuscular fatigue during hypoxia is mediated by the hypoxic ventilatory response

**DOI:** 10.1186/2046-7648-4-S1-A41

**Published:** 2015-09-14

**Authors:** Geoffrey Hartley, Cody Watson, Philip Ainslie, Matthew Greenway, Stephen Cheung

**Affiliations:** 1 Environmental Ergonomics Laboratory, Department of Kinesiology, Brock University, St. Catharines, Ontario, Canada; 2 University of British Columbia Okanagan, Okanagan, Kelowna, British Columbia, Canada; 3 McMaster University, Hamilton, ON, Canada

## Introduction

Neurons of the corticospinal tract are inherently sensitive to oxygen availability and, in response to hypoxia, reduce their metabolic requirements and activity [1]. Consequently, hypoxia is associated with neuromuscular fatigue, attributed in part to central (i.e., CNS) mechanisms [2]. Although changes in cerebral blood flow (CBF), mediated by the ratio of hypoxia induced vasodilation to hypoxic ventilatory response (HVR) induced hypocapnia (i.e., PETCO_2_) [3], may be implicated in the development of central fatigue, the contribution from the chemoreflex control of HVR and CBF vs. reductions in CBF *per se *has yet to be isolated.

## Methods

Neuromuscular function, indicated by voluntary torque production, motor evoked potentials (MEP), M-waves and cortical voluntary activation (cVA) of the flexor carpal radialis muscle during isometric wrist flexion was assessed (n = 8; 27 ± 8 y) during 3 separate conditions: 1) poikilocapnic hypoxia (Poikilo); 2) isocapnic hypoxia (Iso); and 3) isocapnic hypoxia and cyclooxygenase inhibition using Indomethacin (Indo) to selectively reduce CBF (estimated using transcranial Doppler ultrasound). End-tidal forcing was used to control P_ET_O_2 _(51.5 ± 5.1 mmHg) during all conditions and PETCO_2 _at eucapnia during Iso (43.4 ± 4.0 mmHg) and Indo (41.6 ± 3.8 mmHg). Measurements were taken during baseline and upon steady-state response (i.e., stable S_a_O_2_) to hypoxia (approximately 5 minutes).

## Results

The experimental conditions successfully isolated CBF and PETCO_2_. Iso and Indo were associated with a pronounced HVR (0.93 ± 0.60 L·min^-1^.S_a_O_2_^-1 ^and 1.15 ± 0.72 L·min^-1^.S_a_O_2_^-1^) vs. Poikilo (0.26 ± 0.15 L·min^-1^·S_a_O_2_^-1^, p < 0.05). Torque was reduced from baseline in all conditions (-10.9 ± 13.7 Nm, p = 0.03). MEP amplitude (% M-wave) decreased in Poikilo (-4.5 ± 3.5%, p = 0.02) and Indo (-4.5 ± 4.8%, p = 0.02) vs. Iso (0.8 ± 2.8%; p = 0.9). No changes were observed in M-wave (p = 0.81). cVA decreased in all conditions (p < 0.01); however, reductions were greater during Iso (-11.5 ± 9.3%, p = 0.01) and Indo (-12.5 ± 9.1%, p = 0.04) vs. Poikilo (-3.8 ± 11.5%, p = 0.77).

## Discussion

Consistent with previous research [2], hypoxia resulted in impaired neuromuscular function (i.e., reduced torque) in all conditions. These reductions were mediated by the CNS, as cVA decreased in the absence of changes in M-wave. Reductions in cVA were greater during Iso and Indo, suggesting an association with the magnitude of the HVR. Reduced CBF during Poikilo and Indo was associated with decreased motor cortex excitability; however, was not associated with decrements in torque or cVA.

## Conclusion

This study demonstrates that the severity of CNS-mediated neuromuscular fatigue during hypoxia is dependent on the magnitude of the HVR, independent of changes in CBF.
